# Cultivating Psychological Resilience of Israeli Medical Directors of COVID-19 Divisions: The Dynamic Spheres of Salutogenics

**DOI:** 10.3389/fpubh.2022.801297

**Published:** 2022-04-14

**Authors:** Gabay Gillie, Lior Naamati-Schneider, Dvora Pikkel

**Affiliations:** ^1^Multidisciplinary Studies, Achva Academic College, Arugot, Israel; ^2^Department of Health Services, Hadassah Academic College, Jerusalem, Israel; ^3^School of Medicine, Ben Gurion University of the Negev, Beer Sheva, Israel

**Keywords:** COVID-19, front-line clinicians, lived experience, medical directors, psychological resilience, salutogenics, thematic analysis, well being

## Abstract

**Purpose:**

There are a few qualitative studies on the psychological resilience of COVID-19 medical directors upon outbreaks of pandemics. Psychological resilience is essential to providing quality care through the pandemic.

**Materials and Methods:**

We conducted narrative interviews with 14 out of 21 medical directors of COVID-19 divisions in Israeli public hospitals upon the outbreak of the pandemic and through its first wave. We adopted the Salutogenic paradigm to identify personal and organizational resources that both deterred and promoted resilience of front-line medical directors. Thematic analysis was performed based on the Sense of coherence construct, an anchor of Salutogenics.

**Results:**

Low comprehensibility was compensated by ethical boundaries and managerial experience. A few organizational and personal resources promoted manageability. The hospital management both deterred and promoted resilience. In contrast to Salutogenics theory, meaningfulness was driven by the occupational calling rather than by comprehensibility and manageability. Gaps in personal resources inhibited resilience.

**Conclusions:**

Our study adds to the scant qualitative research performed upon the outbreak of the pandemic and extends the Salutogenic paradigm suggesting that the three axes of sense of coherence are multi-layered, intertwined, and evolving. We introduce the dynamic spheres model that we adopted from Physics to illustrate the findings. We propose interventions to build resilience in front-line medical directors.

## Introduction

Since the COVID-19 outbreak, clinicians have been reporting heightened stress, depression, anxiety, hopelessness, helplessness, and fear (1, 2). The fear due to the unfamiliar disease and to making clinical decisions without a protocol, has been associated with psychological distress, poor physical health, and risky health behaviors decreasing immunity (2, 3). Clinicians felt obligated to work around the clock, struggling to balance their own physical and mental needs with those of their team members (4). More responsibilities, a busy work schedule, and emotional exhaustion jeopardize clinicians' health (5). In addition, clinicians experienced ethical conflicts between their responsibility to care for the ill and their right to protect themselves from a deadly virus (6). Clinicians were distressed about discontinued supplies of limited equipment (7).

Moreover, clinicians experience discomfort during endless shifts during which they wore goggles, N95 masks, and full body protective suits, that exacerbated their exhaustion and limited their hearing, causing communication barriers with team members and patients (8, 9). Furthermore, clinicians could no longer rely on facial expressions in communication and had to learn to interpret eye expressions as the sole non-verbal form of communication (10). Since clinicians may potentially spread COVID-19 and feared of infecting family members, they stayed away from their families (11). When they didn't distance from their families, they experienced stigma in their own home communities, causing them frustration and anger (8). Fifty six percent of clinicians treating COVID-19 patients, presented symptoms of posttraumatic stress disorder 58.6% reported an anxiety disorder, and 46% reported depression (12).

Resilience is a process of adapting, and withstanding adversity (13, 14). Resilience refers to a “rebound ability,” alleviating the adverse effects of stress (15). Psychological resilience is negatively associated with depression, anxiety, irritability, and burnout (2, 12). Resilience results in better coping, better health, higher well being, and higher productivity (16). Resilience capacity exists in all people but varies between individuals, depending on personality, interpersonal and social background (17). Resilience may be strengthened by using personal and organizational resources that reduce the impact of traumatic events, preventing a post-traumatic stress disorder (14). While clinicians treating COVID-19 patients play a critical role in global and national health, they constitute the group with the lowest psychological resilience and their psychological resilience is not prioritized (12). Poor resilience leads to negative emotions that further compromise the mental well being of clinicians and inhibit the integration of self-regulation, goal setting, and effective decision-making, essential to effective responses while providing patient care (2, 12). Psychological resilience is essential for frontline-clinicians but the conceptualization of resilience of clinicians is premature and scattered (18).

Although qualitative research has much to contribute to the understanding of the unique and complex experiences of medical directors, qualitative studies addressing experiences of front-line medical directors of COVID-19 divisions upon the outbreak and during the crisis, are scant and retrospective (2, 19, 20). Quantitative studies identified personal resources that promote psychological resilience among front-line clinicians in COVID-19: Less worry about being infected, higher life satisfaction, optimism, social support, avoiding information overload, and a sense of control over adversity (12). It is challenging to conduct rigorous qualitative research with clinicians already struggling with patient care during a crisis outbreak and provide actionable insights from qualitative studies in a timely manner (21, 22). However, a better understanding of personal and organizational resources that cultivate resilience of front-line clinicians during a health crisis may direct efforts to build psychological resilience during the next waves of the pandemic and in future crises more effectively (23). This qualitative study responds to previous calls exploring the cultivation of psychological resilience of frontline-clinicians at both the individual and the organizational level during a pandemic (2, 11, 24). We explored the lived experiences of front-line medical directors upon the outbreak of the COVID-19 in March 2020 through June 2020, in Israel, aiming at gaining insights on how medical directors responded to the pandemic, at identifying factors that cultivate resilience, and at suggesting interventions to mitigate the negative impact of COVID-19 and cultivate psychological resilience.

### The Theoretical Framework of Salutogenics

The current study adopted the salutogenic paradigm which explains successful coping with stressors and adjustment and functioning in adults who face adversity (25). Salutogenics views health as a continuum ranging from “total absence of health” to “total health” (26). Movement along this continuum is initiated when people are confronted with stressors that disturb their homeostasis in their internal or external environment (26). Those who successfully manage stressors are on the health side of the continuum avoiding traumatic experience, while those who are unable to manage the stressors move toward the “dis-ease” side resulting in poor resilience, psychological disorders, and post-trauma. Salutogenics posits that the world is complex and uncertain, and stressors are challenges that may lead to health and growth among those who function effectively in adversity (27). A central concept in salutogenics that facilitates effective coping with adversity is ‘Sense of Coherence' (SoC) (27).

Individuals with SoC adapt and become resilient in the face of life's obstacles; they have a positive and productive attitude enabling them to understand and meet complex challenges (28). They are consistently and enduringly confident that the stimuli deriving from their internal and external environment are structured, predictable and explicable; that they are able to meet demands with the resources available to them; and that these demands are worthy of engagement. SoC is the trust that the challenge is understood (comprehensibility), and the belief that the available resources suffice for coping (manageability). The strength of the SoC is determined by one's choice to adapt to adversities even when available resources are insufficient (14).

The three axes of SoC are: (a) Comprehensibility, (b) Manageability, and (c) Meaningfulness (26). *Comprehensibility* is one's cognitive ability to find logic in multi-adversity situations and view them as orderly, coherent, clear, and structured. *Manageability* is one's ability to cope and resolve problems using skills and resources that facilitate control of the situation. Comprehensibility and manageability create meaningfulness, i.e., one's ability to find emotional meaning in challenges and feel that actions have a subjective, positive meaning that makes sense in life (26). These three axes orient people toward the resources available to them in multi-adversity. People with a stronger SoC are better able and more highly motivated to cope if they understand the stressors (i.e., comprehensibility), select an appropriate strategy and marshal resources to deal with the stressors (i.e., manageability), and have a stronger feeling that engaging with the stressors is a meaningful process (i.e., meaningfulness) (29).

While the salutogenic paradigm is starting to impact theory and research in healthcare, it is understudied (27). Thus far, studies on salutogenics in health have mainly focused on public health and patients in the community while they are sparse regarding clinicians in general, and particularly clinicians in a health crisis (14). Since years of salutogenic research demonstrate that SoC is a powerful explanatory factor of health outcomes, coping, and resilience, salutogenics may be applied for the benefit of clinicians as well (30). We seek to provide new theoretical insights regarding resources underlying the coping of clinicians during COVID-19 and shed light on potential interventions to build and maintain SoC. This study aims to identify personal and organizational resources that facilitated psychological resilience upon the COVID-19 outbreak among medical Directors. The Research Questions Are: (a) What Were the Experiences of Front-Line Medical directors? (b) What were the organizational and personal resources that facilitated their adaptation and functioning as they managed the COVID-19 divisions during the pandemic? (c) What was the role of the resources in the three axes of SoC? (d) What recommendations may be derived for interventions to build resilience?

## Materials and Methods

### Ethics

The board of ethics in the academic institute with which the second author is affiliated granted ethical approval for this study. All participants signed a digital informed-consent form regarding participation and publication before beginning the interview.

### Sample

Participants were senior physicians specializing in infectious diseases, emergency medicine, and intensive care who were assigned as medical directors of the COVID-19 divisions at 21 Israeli public general hospitals from March 2020 to June 2020. Fourteen (12 males and 2 females, ages 46 to 55) out of 21 division directors participated.

### Procedure

We faced acknowledged challenges of data collection in qualitative research during a health crisis and were unable to conduct face-to-face interviews (31). Our responsibility as qualitative researchers, however, was to study the lived experiences of front-line clinicians upon the outbreak of the crisis rather than retrospectively. We adopted a digital, internet-based method (ZOOM videoconferencing platform) for data collection as a recourse for conducting our research (32). Since virtual interaction may alter relationships, we applied participatory research by conducting trial interviews with three directors from private hospitals, to test whether ZOOM can serve as a social form of interaction (33). Based on their reports we concluded that using ZOOM does not compromise effective communication with interviewees, although paying heed to body language through the ZOOM is challenging.

Following ethical approval, we collected names of COVID-19 division directors, obtained their phone numbers, and sent them messages asking if they are willing to participate in a study on the experiences of directors of COVID-19 divisions. Fourteen out of 21 directors agreed to participate. We were either unable to track the phone number of others or received no response to our messages. Upon receiving their consent, we held a short phone conversation with each participant to introduce ourselves, explain the study goals and methodology, and ask them to minimize the disturbances during the interviews. We assured them that their participation would be anonymous and confidential, and that we would conceal any information possibly identifying them or their hospital (34). Considering the time constraints of interviewees, we scheduled interviews per their requests. We informed interviewees that they could stop the interview at any point they choose, and interviewees acknowledged their understanding that parts of their interview will be published (34). A link to the interview was sent a week in advance. No personal data was entered into the invitation. To assure confidentiality, we canceled the Zoom recording function and recorded the interviews using an external recorder. Only invited participants could enter the ZOOM meeting using a password. We used the video to facilitate natural interaction during the interview. A wide bandwidth, adequate lighting, and the quality of the participants' video cameras enabled smooth interviews. Non-verbal facial responses were evident (19).

We held 14 45-min narrative interviews. We worded the one general open-ended question to encourage participants to share a deep, unstructured narrative (35): “Please tell me about your experience as a COVID-19 division director since the first COVID-19 patient arrived until the time of the interview.” Most interviewees responded with silence and commented that it is a complex question. However, once they started sharing their experience, there was no need to elicit further details nor to amplify their answers; the barriers fell, and additional questions were not necessary (35, 36). During the interviews, there were moments of silence, perhaps enabling the interviewees to process their thoughts and feelings. We made no attempt to comment, ask questions, or judge what participants said. After audio-recording interviews were transcribed. Following data analysis, we translated the findings from Hebrew to English.

### Data Analysis

We performed thematic analysis, a qualitative method that fits well with our epistemologies, our theoretical anchor, and our research questions for identifying, analyzing, organizing, describing, and reporting the themes within the data (37). Thematic analysis is effective for exploring the perspectives of the interviewees, for highlighting similarities among them, and for generating unanticipated insights (38). We familiarized ourselves with the data, generated initial code description using coding by the three SoC axes of comprehensibility, manageability, and meaningfulness, allowing us to simplify and focus on the Salutogenic characteristics of the data (26, 39). We searched for themes in each axis and reviewed the themes. Data analysis was an iterative, reflective process that developed over time, involving constant moving back and forward between phases and weekly meetings between the researchers.

We generated themes and categories that conveyed the interviewees' meaning and identified links between the themes; produced a list of main themes which captured the interviewees' main concerns; and presented evidence in words from the interview. In line with theory-based analysis, the dynamic sphered model emerged from the data itself and captures them (**Figure 4**). Elements derived from patterns such as recurring meanings and feelings were marked as themes (37). By bringing together elements of experiences, which are often meaningless when viewed alone, we made sense for the specific context of this study. Themes and behavior patterns emerged from the data through six analytical steps: (1) We independently read and re-read the interviews and listed patterns of experiences through direct quotes. (2) We then identified all data that related to the patterns already classified. (3) We sorted all data according to the corresponding patterns. (4) We combined and categorized related patterns into sub-themes to obtain a comprehensive view of the emerging patterns. (5) We pieced together themes in a meaningful way to form a comprehensive picture representing participants' interpretation of their coping experience (37). (6) By referring to the literature, we obtained information that allowed us to make inferences from the interviews regarding resilience.

### Quality Criteria

To ensure that our findings are relevant and actionable we collected data in real time rather than retrospectively. We generated data in a short window of time with a fast recruitment and extensive data collection (40). We were transparent and disclosed the study purpose and rights of the participant. We asked how they feel, to create feelings of connectedness (33). We attempted to make participants feel comfortable as they shared their narrative. The interviews revealed unanticipated themes, facilitating an in-depth understanding of the reality of medical directors from their perspective in a very extreme health crisis at its initial outbreak. The unstructured narrative interviews relied on the interviewee's subjective, spontaneous responses to the question enabling us to understand their perceptions without imposing any prior categorization which might narrow our field of inquiry (35).

We analyzed data using provisional coding guided by the dimensions of Salutogenics as categories and explored data to identify themes (37). To assure reliability, we analyzed all interviews, identified themes and subthemes in the data independently. This study was not initially based on Salutogenic theory, but during the data analysis stage we realized that the theory of Salutogenics may be related to the emergent themes and adopted this theoretical framework. Therefore, SoC wasn't measured directly by specific questions but rather examined according to the three axes of SoC that emerged from the interviews: comprehensibility, manageability, and meaningfulness. Since SoC was not measured, findings do not reflect a change in SoC. Findings reflect the evolvement in the way that directors of COVID-19 divisions perceived themselves, the challenges and the resources that deterred and promoted resilience. Findings reflect processes and outcomes as in previous studies that analyzed the three axes of SoC in different contexts (41–43).

Qualitative research encompasses the perspective of the researchers rather than objective reality. As the human instruments making judgments about coding, theming, decontextualizing, and recontextualizing the data, we ensured the coding creates trustworthiness through credibility, transferability, dependability, and confirmability (44). We recorded the study logistics, our methodological decisions, our personal values, our reflections, and insights after each interview (44). We believe coping is modifiable and that stressing resources that enable coping may improve resilience in the face of adversity.

## Results

The themes we identified revolve around the three axes of SoC. We present factors deterring SoC and factors promoting SoC in congruence to its three axes (26). In parentheses we present the percentage of interviewees who shared the presented theme. In bold we present what interviewees highlighted.

### The First Axis of SoC: Comprehensibility

**Figure 1:**
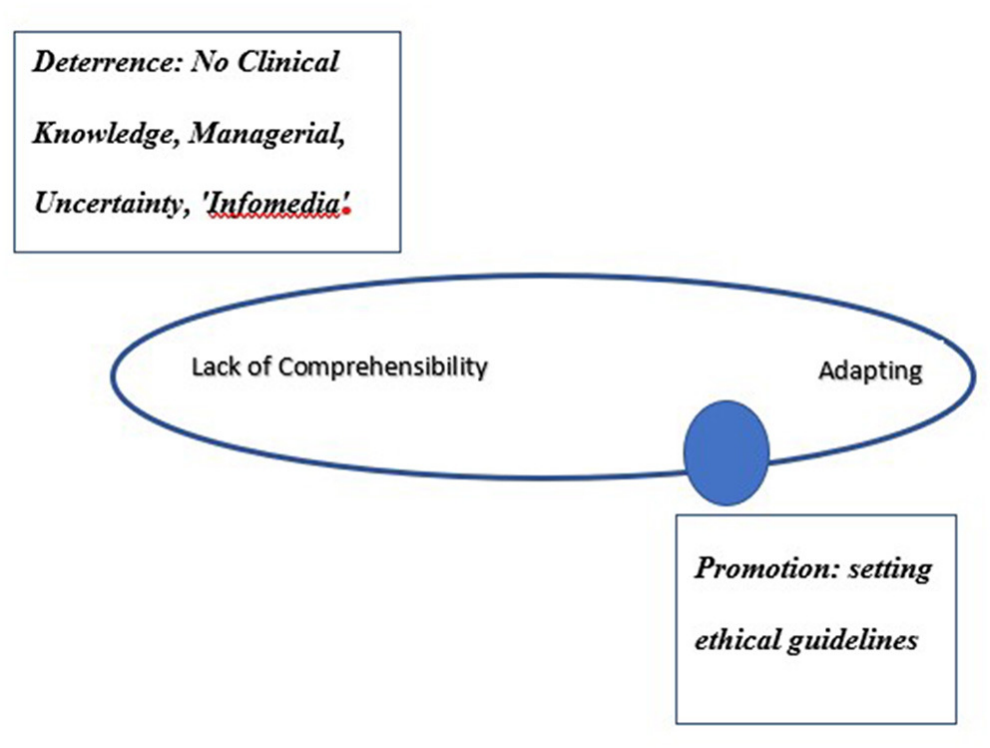
The dynamic sphere of resilience on the comprehensibility axis.

Findings present the level of a dynamic feeling (or lack of feeling) of confidence that the stimuli deriving from one's internal and external environment are structured, predictable, and explicable. All interviewees referred to the high uncertainty, lack of familiarity, lack of knowledge about COVID-19, its attributes, what lies ahead, and giving treatment without a protocol. Directors referred to two points in time: the initial encounters with COVID-19 patients during the first 3 weeks and encounters with COVID-19 patients thereafter. They all described an evolving dynamic experience. Figure 1 presents the sphere of resilience on the comprehensibility axis starting from lack of comprehensibility to adapting.

### Resilience Deterrence

#### Lack of Clinical Knowledge (100%)

Interviewees described their experience of helplessness, frustration, and speculation about the exceptional situation they found themselves facing:

“*The virus itself was new to medical systems, at the beginning, **we knew nothing about this disease**. How it attacks the body, how it passes from patient to patient, how it can be defeated. **Not to know a disease in 2020 is an unusual medical situation***.” (2, Female); *“I am a senior expert, and I realize that **I have never** seen anything behaving the way this virus is behaving*. (10, Female).”

Interviewees were troubled by the fact that in contrast to their core professional value, they were treating patients without a protocol, eliciting a sense of guilt and helplessness in the first 3 weeks*:*

“*It was very stressful. We knew we did not know much about the disease and we were going to treat patients with no protocol. Everything was changing from day to day.”* (4, Male); “*For the first 3 weeks we did not see where the disease was going, and how to treat it*. (8, Male); “*It is disturbing that our actions are not necessarily the right ones for the patients*.” (Female, 10).

#### Managerial Uncertainty (100%)

Interviewees faced new challenges and expressed their frustration and fear**:**

“*I did not know how to act with people who do not want to work in the COVID division.”* (10, Female)*; “We did not know how to communicate with patients and with their families”*. (6, Male)*; “It took a few days for each of us to process the experience of entering the ward. I was terribly scared”* (13, Male)

#### A Flood of Information (100%)

The overwhelming flood of information was perceived as necessary but terribly frustrating:

“*I call it **'Infodemia**': a huge outpouring of information about the virus that was very confusing and full of contradictions. The information reflected the nature of the virus: confusing, deceptive, and constantly changing. **Virality of information transfer**” (2, Male); “Information from Europe kept coming with horrifying scenarios”* (14, Male).

### Resilience Promotion

#### Setting Ethical Boundaries: (30%)

Half of the interviewees described the process of setting clear ethical boundaries as an important facilitator of coping:

“*At first, we gave patients different compassionate drugs but at one point we decided that we are not doing compassionate therapies and not doing science fiction anymore. We decided we would do nothing more without evidence and it made us recognize our limitations, **it was, very, very helpful** (1, Male); “Many families asked, “Why don't you give plasma?” or “Why do you do it this way?” I decided to explain to families that what they heard is not applicable, that it may be good for YouTube but will not help their loved one. It worked; we do not get carried away because of the panic around us.”* (4, Male).

#### The Second Axis of SoC: Manageability

**Figure 2:**
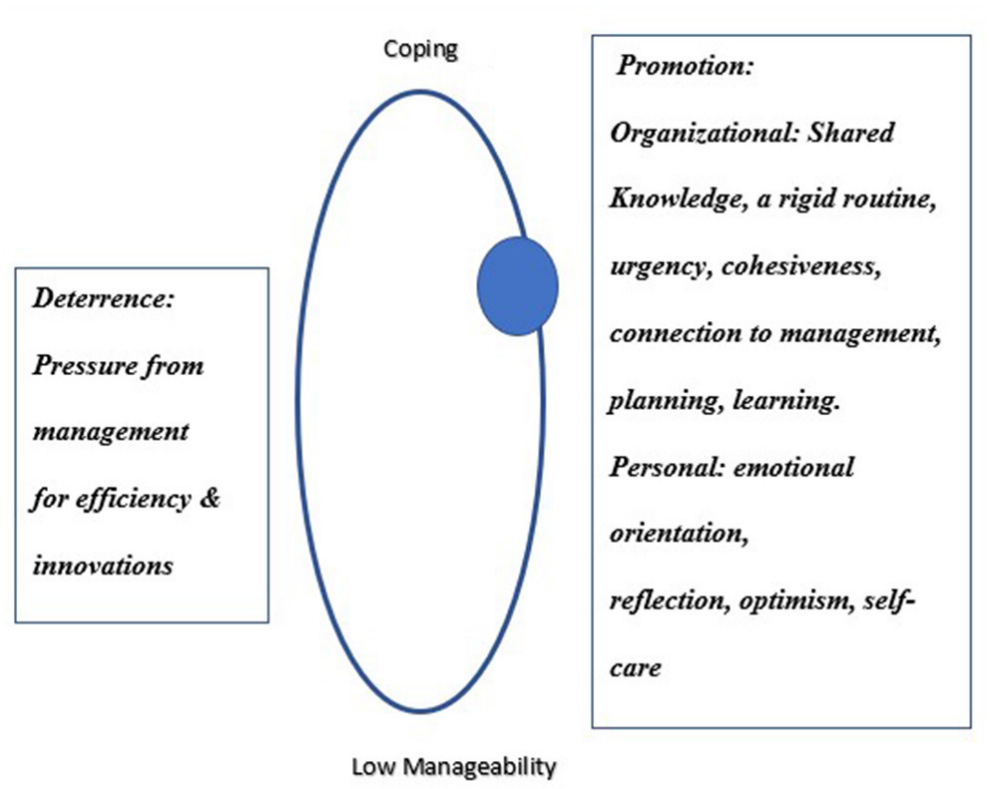
The dynamic sphere of resilience on the manageability axis.

Findings present the personal and organizational resources in clinicians' internal and external environment and their role as deterring or promoting manageability in meeting the demands posed by the COVID-19 outbreak. Figure 2 presents the sphere of resilience on the manageability axis, moving from poor manageability to coping.

### Resilience Deterrence

#### Pressure From Management to Be More Efficient and to Apply Innovations (40%)

*I asked my management “Do you really want someone to resuscitate two patients with one ECMO?”, I was not willing, I said, “Find someone else”. I went through a process of understanding what I am and am not willing to do, despite the pressure and tension …”* (8, Male); “*So many inventors and entrepreneurs came here to test their machines or 3D printer masks or filters for the machines…some clinicians tried innovations…As if in any other situation, they would roll in, go up the stairs, skip 3–4 years of experimentation, and **skip a Helsinki approval** before approaching a patient. At one point I did not allow it anymore.”* (12, Male).

### Resilience Promotion

Manageability entailed organizational processes (sharing, assigning interns, cohesiveness, teamwork, sticking to routine, and connection to management); professionalism (accountability and learning); and inner resources (emotional orientation, self-care, reflection, and optimism).

### I. Organizational Resources

#### Knowledge Sharing (100%)

Interviewees reported that resources of time and attention were invested in sharing knowledge as an ongoing process during the crisis:

“*Everyday doctors all over the world learned something new and shared it*” (6, Male); “*We held daily meetings. The decisions were joint, and the response was complete and swift. It provided a sense of security.”* (9, Male); “*It was clear that what was happening to me may not happen to others so we must each share our experience amongst us and with the world*.” (11, Male).

#### Sticking to a New Routine (100%)

Interviewees sustained the routines of morning visits, professional discussions, and learning during the crisis:

“*I produced regular morning sessions at 8 A.M. It is no different than what we do on a daily basis but it's at a different scale, level of strain, and numbers*.” (13, Male); “*We have a very rigid pattern of morning visits, then relaying information to the senior doctor in charge, then joint discussions about patient needs.”* (12, Male).

#### A Sense of Urgency and Team Cohesiveness (100%)

Interviewees described the importance of the organic team and the cohesiveness that was created although the teams were no longer organic:

“*Working with your organic staff is very central in such situations. If you build the team the right way, you produce a system that works right. I had to protect clinicians who are at a greater risk.”* (10, Male); *“It seems to me that we won the COVID-19 due to treating COVID-19 as a draft notice. Everyone is drafted, everyone helps”* (4, Male).

Interviewees described the individual contributions of each team member, the sense of urgency, and the removal of boundaries between medicine and nursing:

“*There is a fighting spirit, everyone is present and connected, the situation went from zero to a hundred very quickly.”* (3, Male). “*When we are together, we have unlimited power* (2, Female)*; “The sense of pioneering and the contribution of each member shapes the division's character. The classic hierarchy of medicine in which we grew up is flattened, the clinical impressions of the nurses don't fall short of those of the doctors.”* (14, Male).

#### Planning and Allocating Residents (80%)

Interviewees allocated tasks according to the strengths of their personnel:

“*Proper management requires the understanding that in the end, the human capital is the heart of the matter” (*8, Male); “*We understand the need to use lots of technologies, so our interns who are good at technology help us with that.”* (5, Male).

#### Connecting to Management (50%)

Interviewees stressed their communication with top management as a fast route to getting required equipment:

“*We talk with management constantly* as *the reference point for everything in this story.”* (9, Male); “*The direct connection to management resulted in getting everything I wanted whenever I wanted it, enabling me to manage macro, not just micro.”* (7, Male)*; “I have the means; bureaucratic issues do not exist. Everything is geared toward optimal patient care.”* (8, Male).

#### Learning (20%)

Interviewees related to the importance of including learning and briefing in work processes in those turbulent times:

“*Every morning we present the studies that are relevant to our patients. We publish, we teach. We constantly consider what we do well and need to improve; we learn very quickly*” (7, Male).

### II. Personal Resources

Interviewees related to personal resources that enabled their adaptation: Emotional orientation; reflective skills; preventing the neglect of self-care, and optimism in the face of horrific scenarios.

#### Emotional Orientation (20%)

Interviewees expressed their fear and elaborated on how it affects their functioning:

“*I worried about how I could meet all the demands of providing care and keep the staff from getting infected. That fear was very noticeable at first.”* (4, Male)*; “The main fear is that you do not know if on the next day you will encounter a catastrophe, if you will be flooded with patients, if the pictures of Italy and New York will be in your backyard”* (11, Male).

#### Reflection Abilities (20%)

Three interviewees were able to distance themselves from the situation, reflect on it, and gain insights:

“*In an emergency you work with people who are not in their comfort zone. You, too, are not in your comfort zone. We are continuously out of our element. It adds complexity, but for me it's partly why I specialize in intensive care……[Quiet]”* (8, Male); “*We learn to step forward out of the fog of uncertainty. We rely on intuition, on gut feeling, and we acknowledge that we do not have all the data. We must feel comfortable with uncertainty.”* (2, Female).

#### Optimism (20%)

Optimism served as a beacon of light as interviewees expected positive changes to take place:

“*People said that my TV interview was a broadcast of cautious optimism. After a week and a half everyone here thought it is the end of the world. Suddenly someone said, guys, if we encounter what happened in Italy, we will do everything to take care of everyone, but we must remember that at some point, this virus will subside*.” (7, Male).

#### Self-Care (15%)

Interviewees shared the need for self-care to maintain good functioning:

“*I sat with my team and saw their crestfallen faces. Then I looked at myself in the mirror and thought “I am the cause of this. Stop, take care of yourself, and then come back to the team with new energy.”* (3, Male); “*Before the pandemic, I did a lot of work on myself. Now I take a break and I go out riding on my bike for a bit. If the pandemic had happened 2 years ago, I do not think I would have behaved the way I do. Not in terms of courage but in terms of caring for myself.”* (13, Male).

### The Third Axis of SoC: Meaningfulness

**Figure 3:**
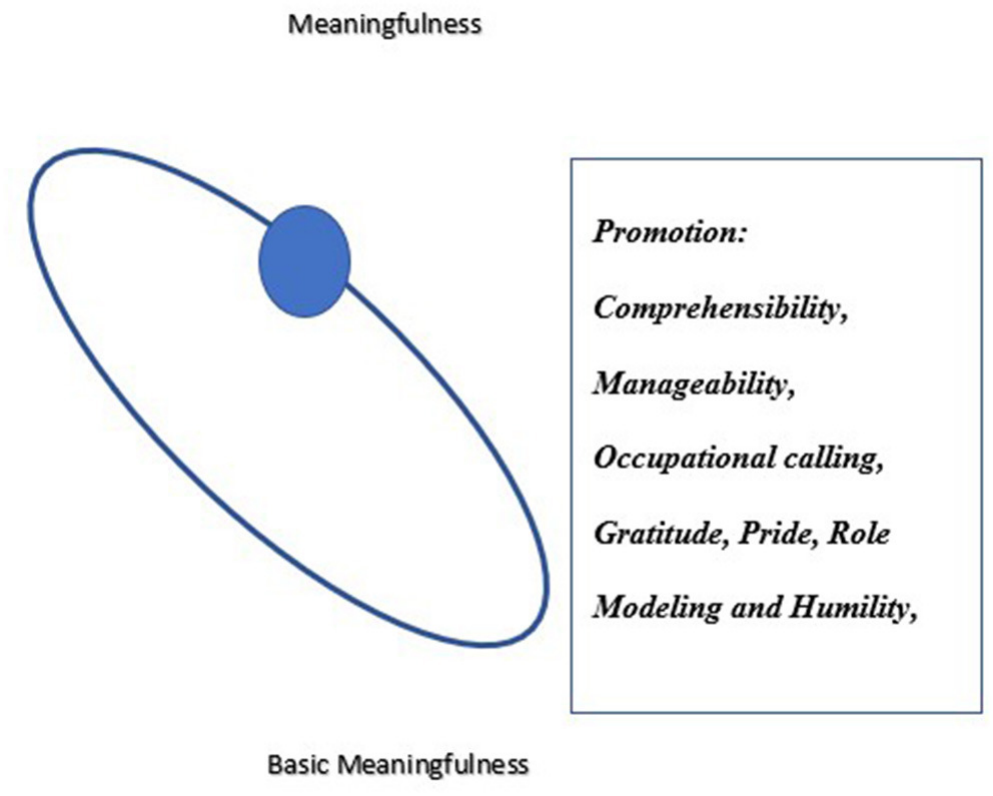
The dynamic sphere of resilience on the meaningfulness axis.

Findings present the emotional axis interviewees perceived as contributing to their individual and collective growth. Meaningfulness was three-fold: as a citizen (national mission), as an expert physician (an occupational mission and professional growth), and as a manager and leader (role modeling, gratitude toward the teams, and humility). Figure 3 presents the sphere of resilience on the meaningfulness axis moving from occupational calling to gratitude, pride, role modeling, and humility.

### I. Fundamental Meaningfulness as a Citizen (100%)

As citizens, interviewees viewed their work in the division as a national mission. They reported feeling “conscripted” from the start of the pandemic:

“*Eventually we realized we were doing something extremely important. When we saw the triumph as patients recovered, we felt close to them, it was very touching*.” (6, Male).

### II. Fundamental Meaningfulness as a Physician (100%)

Interviewees shared that their assignment as managers of the COVID-19 division is a once-in-a-lifetime opportunity, and they reported on their professional growth**:**

“*It is challenging, very interesting period, really unforgettable. It's heartwarming. There has never been such value to my work as there is now. I always feel the power of a kind of sacred work. Everyone is expecting us to reach a minimum of deaths and a minimum of side effects. Today I can proudly say that we could really stand up to this difficult task.”* (9, Male); “*It is a very different and very special experience, I love the challenge, I like to be at the forefront of the action. I pushed myself hard into this place. This is the essence of being an intensive care physician*.” (14, Male)

### III. Meaningfulness as a Manager and a Leader (100%)

#### Gratitude and Pride (40%)

Interviewees shared that the sense of meaningfulness as managers includes gratitude toward team members and remembering that as leaders, they must be role models:

“*I'm very proud that the clinicians, including some interns returning from rotations, showed maturity, great commitment to the task. I am really proud of the team. We worked a lot of hours, no days off on Fridays, Saturdays, no Passover, no Independence Day…”* (11, Male)*; It is disturbing that our a “Thanks to my wonderful team members, who once again proved their abilities, we met this challenge.”* (Male, 14).

#### Role Modeling (70%)

Directors shared that they were aware of their influence on clinicians in the division and their deliberate efforts to serve as role models:

“*My leadership definitely affects how people work. When the leader is charismatic, it makes a difference. I am not attesting to myself, God forbid, but I am giving this as an example of what contributes”* (7, Male); “*With my team, the essence of my role is fatherly. Even when I have my fears, I have to show a lot of courage.”* (12, Male); “*As division manager, I had to lead the way* (9, Male).

#### A Lesson in Humility (20%)

Three interviewees related to the pandemic as a reminder of how fragile we all are and of our inability to control our world:

“*Who is a wise person? A humble person. One who is willing to hear from every person and even from the least important of all, one who lowers himself and listens to others. It is the knowledge that not all powers are concentrated in my hands and there are things that are more important than me”* (3, Male); “*The virus brought humility; as senior experts, it made us hear all the voices because only together could we find the right way to treat the disease.”* (2, Female).

**Figure 4:**
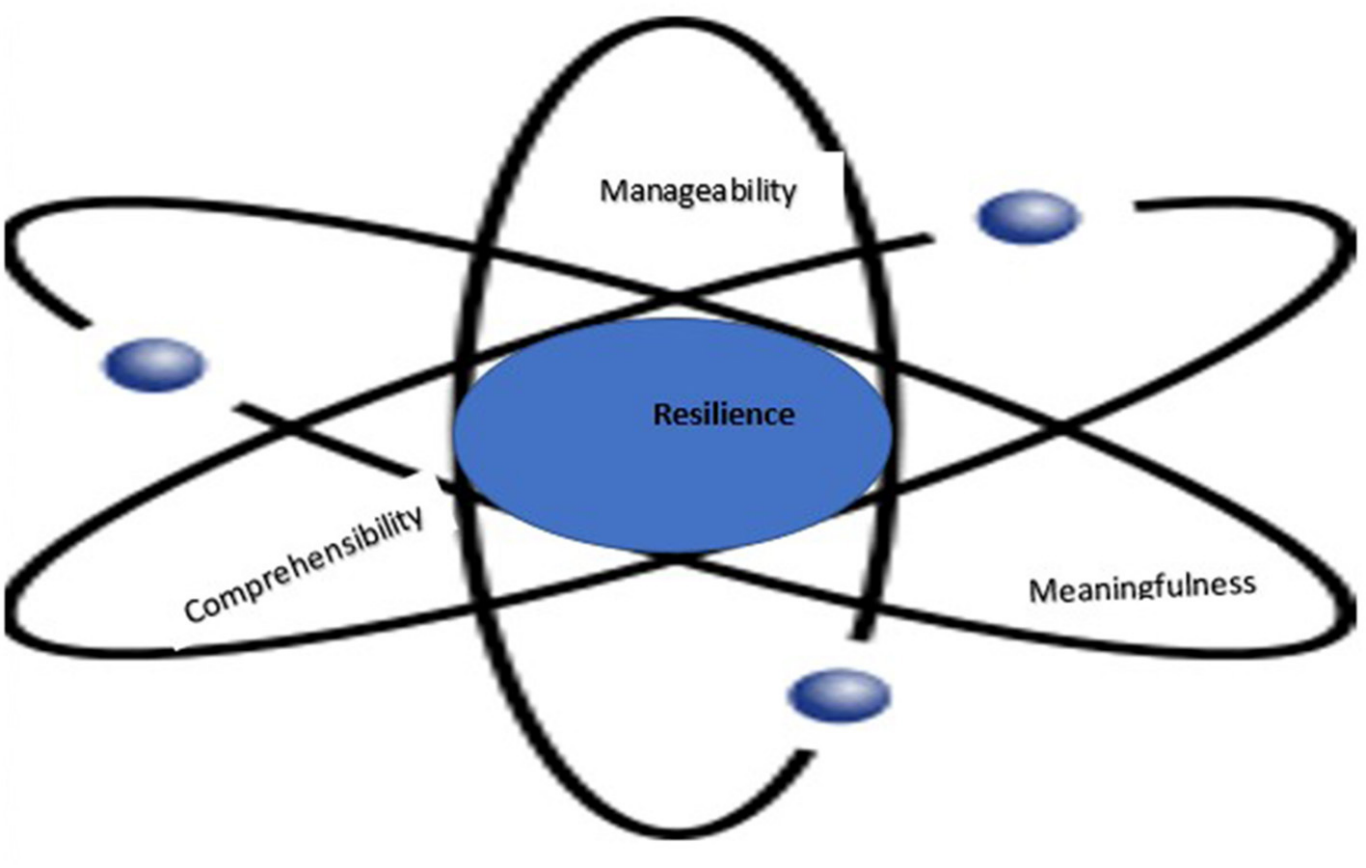
The dynamic spheres of resilience model.

Data conveyed SoC as evolving around themes that promote each of the three axes, revealing a salutogenic model that affected collective SoC as well. Figure 4 presents the data-based *Dynamic Spheres Model of Resilience* illustrating the complex intertwining of the three multi-layered axes creating psychological resilience. This innovative model presents the axes as dynamically revolving around the resources that both deter and promote resilience. The model is derived from physics and is based on the classic atom shape with a nucleus in its center and spheres around it, all in constant motion. On each of the spheres the “nucleus” moves between their poles: from poor resilience to resilience. The model presents a different trajectory for each axis.

## Conclusions

This narrative study is the first to fill the gap in the state of the art, exploring the lived experiences of directors of COVID-19 divisions applying salutogenics to identify resources that promoted their psychological resilience. Participants reflected upon the resources they developed, the actions they took, the lessons they learned, and the reasons they keep going. They shared their personal and managerial voice reflection on their coping as they faced a novel complex situation. Antonovsky (26) maintained that the SoC is a unidimensional construct, with a global orientation. Other scholars argued that it is tri-dimensional, establishing the three interrelated but separate axes of SoC (14, 27) This study extends the Salutogenic theory demonstrating that in a pandemic, resilience of front-line directors in each of the SoC axes is not only tri-dimensional but also multi-layered, evolving, dynamically operating among the layers of each axis, entailing resources that both deter and promote SoC. Also, the three axes are intertwined enabling adaptability that evolved over a short time, from lack of comprehensibility, to using personal and organizational resources to manage the situation, through meaningfulness.

### Comprehensibility

Participants lacked an understanding of the disease, were flooded with contradicting information about it, and were confused yet needed to provide care without protocols. The lack of comprehensibility was a deterring factor for resilience, decreasing self-efficacy and creating helplessness and fear. Within 3 weeks, medical directors compensated for their lack of clinical comprehensibility through their managerial and clinical experience. The multi-layers of comprehensibility as physicians, as managers, as supervisors, and as leaders, may compete amongst them as they all require mental resources and energy. A daily prioritization among competing layers created clarity and facilitated effective functioning. It also may have balanced the tension between the multi-layers, facilitating manageability of the chaos. Only half of the participants reported setting clear ethical boundaries as a game changer that facilitated the development of self-efficacy, conveying their recognition of their past accomplishments. Findings suggest that conflicting layers of comprehensibility existed simultaneously. Despite the new challenging circumstances, participants reported that it was their experience rather than comprehensibility of the disease that facilitated manageability. Comprehensibility has been thus far viewed as one-dimensional, but findings contradict the theory, suggesting that comprehensibility may not be limited to the scope of clinical understanding but rather encompass multi-layers.

### From Comprehensibility to Manageability

Participants relied on their managerial experience to map challenges and to decide how to best manage the complex situations they encountered. They took responsibility for clinicians who belong to their organic teams but are at high risk for infection. Despite the shortage in clinicians, participants decided to reassign them to units that were safer for them. Participants were challenged by how to respond to clinicians who were afraid of getting infected and refused to work in the COVID-19 division. Another challenge was how to contain the responses of fellow team members, who perceived participants as potential disease carriers and related to them with suspicion and alienation. Participants consciously served as role models, engaging in endless clinical work, and expressing acceptance and caring for clinicians in their division. An in-depth examination of the themes revealed that organizational resources both promoted and decreased resilience. Hospital managements deterred resilience when they expected clinicians to use scarce equipment more efficiently and to use untested innovations, creating moral conflicts and distressing participants. Such lack of perceived support jeopardizes self-efficacy and problem solving of clinicians during the pandemic (45).

Hospital managements promoted resilience when they recruited and directed resources to the COVID-19 divisions. Participants' expertise in saving lives under intense pressure in intensive care and emergency medicine facilitated manageability across work processes, despite the lack of clinical comprehensibility. Directors created rigid daily routines (e.g., morning visits, meetings, seminars) and engaged in shaping procedures that they manage during routine times, perhaps providing a sense of security and supporting their assessment that they are providing high quality care. Participants systematically analyzed what they would need “down the road” and prepared for it rather than focused only on the “here and now.” Participants made sure that their managements understand what must change to put limited resources in place so the division would be ready for the difficulties ahead. Participants orchestrated processes of information sharing, team cohesiveness, and decision making. Personal resources of emotional orientation, reflection, and optimism, self-awareness, awareness of others and empathy, were important in strengthening SoC, but only a few participants harnessed them. The multi-layers of manageability strengthen the individual SoC of the participants. Data analysis regarding the functioning of the COVID-19 divisions, despite the multi-adversity, suggests that the individual SoC of the participants may facilitated a collective SoC of the division.

### From Comprehensibility and Manageability to Meaningfulness

While meaningfulness was theorized as driven by comprehensibility and manageability, for clinical directors it was driven by the occupational calling and was tri-layered. The fundamental layers of meaningfulness as citizens and as physicians contradict the salutogenic theory, as they do not emerge from comprehensibility and manageability. Meaningfulness for clinicians in a pandemic may be atypical to meaningfulness according to salutogenics and may emerge from the chosen occupational calling of saving lives. Contradicting salutogenics, comprehensibility and manageability may strengthen the fundamental meaningfulness among clinicians rather than create it. Participants were able to view the COVID-19 as a once-in-a-lifetime endeavor; they felt passionate and viewed the crisis as facilitating their professional growth. Their self-esteem and self-efficacy promoted meaningfulness and strengthened psychological resilience (46). Meaningfulness as managers and leaders, the third layer, encompasses the gratitude toward the teams and the expression of pride and humility, enhancing participants' own well being and that of team members (47). The capacity to cope infused a sense of triumph, despite all odds, in the face of a virus that keeps striking around the globe.

To sum, this study extends existent knowledge, suggesting that SoC is a multi-layer concept among front-line directors in a pandemic: (a). Comprehensibility is multi-layered. The experience of participants as directors, leaders, and mentors, compensated to a great extent for poor clinical comprehensibility. (b). Manageability, emerging from multi-layered comprehensibility, is also multi-layered entailing managerial experience in shaping organizational and personal resources. (c). Meaningfulness is tri-layered, growing from the fundamental meaningfulness as citizens and as physicians committed to saving lives to meaningfulness as directors and leaders in a crisis, emerging from comprehensibility and manageability. Resources that promoted psychological resilience were personal (ethical boundaries, reflection), organizational (infrastructure support, a sense of connection), and existential (sense of meaning and purpose). Interventions to promote resilience should target both the individual and organizational level (48). Additional personal resources we identified were reflective abilities, self-awareness, empathy, and social skills. These skills are elements of emotional intelligence, directly related to resilience (49, 50). This study contributes to actionable development of resources and capacities that are required for building resilience of clinicians in health crises (23).

### Practice Implications

This study illuminates the need to prioritize psychological resilience among front-line directors in hospitals during crises (51, 52). Given that SoC plays a vital role in the psychological resilience of individuals, we call upon hospital managements to strengthen the SoC of front-line clinical directors, particularly when comprehensibility is low. To promote resilience, managements are called upon to identify gaps in resources that harness resilience and strengthen them through mentorship. To encourage an optimistic attitude and self-efficacy in coping, clinicians need to share their coping behaviors with others (14). An organizational discourse of peer support in chaotic times may establish a network of clinicians with whom to share experiences. Identifying, selecting, and using available organizational resources while adapting to adversity will facilitate more effective responses of clinicians (53). Management is called upon to assign a designated mental health professional to improve the buffering of stress (54, 55). Managements to structure ongoing discussions on significant ethical concerns that may arise during a crisis to develop ethical awareness, support real-time ethical reflections, safeguard clinicians, and enhance their well being. Compromising the psychological resilience of clinicians may constrain personal and professional growth. The psychological resilience of medical directors through a crisis is a prerequisite for their ability to keep caring for clinicians and patients, and to mentor others who depend on their leadership.

### Limitations and Directions for Future Studies

The cultural attributes of interviewees may have influenced the resources and patterns. There may also be failures in picking up on non-verbal cues not visible via the ZOOM interview. Future studies may replicate this study in other countries to explore the perspectives of front-line medical managers on deterring and promoting factors for psychological resilience in health crises.

## Data Availability Statement

The original contributions presented in the study are included in the article/supplementary material, further inquiries can be directed to the corresponding author/s.

## Ethics Statement

The studies involving human participants were reviewed and approved by the Board of Ethics, Hadassah Academic College, Israel. The patients/participants provided their written informed consent to participate in this study.

## Author Contributions

GG: conceptualization, data curation, literature review, data analysis, and writing the first draft. DP: pilot study and review od draft. LN-S: data curation, data analysis, and review of draft. All authors contributed to the article and approved the submitted version.

## Conflict of Interest

The authors declare that the research was conducted in the absence of any commercial or financial relationships that could be construed as a potential conflict of interest.

## Publisher's Note

All claims expressed in this article are solely those of the authors and do not necessarily represent those of their affiliated organizations, or those of the publisher, the editors and the reviewers. Any product that may be evaluated in this article, or claim that may be made by its manufacturer, is not guaranteed or endorsed by the publisher.
